# An Empirical Study of a Pedagogical Agent as an Adjunct to an eHealth Self-Management Intervention: What Modalities Does It Need to Successfully Support and Motivate Users?

**DOI:** 10.3389/fpsyg.2019.01063

**Published:** 2019-05-09

**Authors:** Mark R. Scholten, Saskia M. Kelders, Julia E. W. C. Van Gemert-Pijnen

**Affiliations:** ^1^Department of Psychology, Health and Technology, Centre for eHealth and Wellbeing Research, University of Twente, Enschede, Netherlands; ^2^Optentia Research Focus Area, North-West University, Vanderbijlpark, South Africa

**Keywords:** embodied conversational agent, pedagogical agent, chatbot, eHealth, e-learning, motivation, self-guided intervention, self-management

## Abstract

Prior research has shown that the more patients know about their disease, health, and lifestyle the better the health outcomes are. Patients who are suffering from either a physical disease with mental consequences or from mental illnesses can contribute to their own feeling of mental well-being by following evidence-based online, self-guided therapeutic interventions. These self-guided therapeutic interventions during which there is no contact with a care provider have shown high effectiveness. However, users (patients) of self-guided eHealth interventions have difficulties fulfilling the entire trajectory as is mirrored in high non-adherence rates. Users have reported a need for support, that is traditionally provided by human care providers. This study investigates the opportunities from within the technology to increase its support level toward the user. We deployed a pedagogical agent acting as an adjunct to a self-guided positive psychology psycho-education intervention. This agent provided instructions and user support in between and explicitly not during the online learning modules as to mitigate the risk of distraction. By setting up a between-subjects design and deploying three versions of a pedagogical agent (also known as Embodied Conversational Agent), varying the features of animation, speech, and visibility we investigated whether users felt more supported than by a fourth text-only control condition. All four conditions provided similar task-related support and emotion-related support to the user. Our results showed that our pedagogical agent made users feel guided and supported with respect to fulfilling their tasks. However, no effects were found of emotion-related support resulting in higher user motivation and an improved learning experience. Significant effects of visibility and voice were found, but animation of our pedagogical agent had no effect. On the feedback outcome variable, we found a gender effect. Male participants graded the *visible* Embodied Conversational Agent (ECA) *higher* than female participants and graded the *non-visible* ECA *lower* than female participants. In our view, ECA’s should not necessarily be deployed with the ambition to compete with the profound human potential to deliver support and guidance. Exploring ECA capabilities merits further attention, from the stance that the technology itself can support users and potentially make them adhere.

## Introduction

eHealth is about the use of information and communication technology to reinforce health and health care. It refers to forms of prevention and education, diagnostics, therapy and care delivered through digital technology, independently of time and place. An important branch of eHealth consists of technological self-care solutions such as home telemonitoring applications that provide patients with direct insights through self-monitored data. Other self-care solutions focus on teaching indirect insights, leading to competence (disease knowledge) or disease management (making choices, acting responsibly) (Peeters et al., 2013). Research showed that the more patients know about their disease, health, and lifestyle the better the health outcomes are (Kennedy et al., 2017). Technological self-care (e.g., for chronic diseases) often goes hand in hand with self-management as a practice: the ability to actively participate in the management of health with the emphasis on physical and mental well-being. This involves medical management; changing, maintaining, and creating meaningful behaviors and dealing with the emotions of suffering from chronic disease(s) (Lorig and Holman, 2003). The question is whether self-management can be independently done by patients, that is without the help and support of a care provider. More precisely, the question is whether patients who are suffering from either a physical disease with mental consequences or from mental illnesses can independently contribute to their own feeling of mental well-being. Meta-analytic studies (Spek et al., 2007; Barak et al., 2008) have demonstrated the effectiveness of self-guided therapeutic interventions during which there is no contact with a care provider. Despite the effectiveness, patients show mixed opinions on these self-guided interventions. On the one hand patients report positive experiences with self-guided interventions (Walsh et al., 2018), but disadvantages have also been reported by patients, such as the lack of human contact (Flynn et al., 2009).

Especially in case of self-guided e-mental Health interventions against depression, adherence can be low (Schubart et al., 2011). Low adherence is sub-optimal as greater exposure to website content is associated with increased benefit (Christensen et al., 2004). Obvious follow-up questions are therefore *why* users do not adhere and especially *how* adherence can be stimulated. There seem to be no final answers to these questions, but cues are certainly available. A meta-analysis of the effectiveness of diabetes interventions suggests that participants’ difficulties in understanding the use of Web-based interventions led to higher non-adherence rates (Lie et al., 2017).

In addition, some studies relate disease-specific effects such as severity to adherence, with a high level of emotional distress leading to early dropout (Davis and Addis, 1999).

In terms of solutions, the provision of support to enable patients to be confident and capable in managing health conditions is generally considered an important factor (Wilkinson and Whitehead, 2009; de Silva, 2011). In addition, there is empirical evidence that the lack of such a supportive relationship is associated with low levels of motivation to engage in self-care and may as such lead to non-adherence (Drench et al., 2007; Bickmore, 2010).

In conclusion, user support is a relevant topic for user adherence. The next question is what kind of support users need. To answer this question, in an earlier study, we have analyzed (Scholten et al., 2017) studies on support needs as expressed by eHealth users. We found that users have a need to be encouraged (emotion-related support) but also value practical support (task-related support). Emotion-related support acknowledges both the user’s endeavors during the change program and the originating issue the user is dealing with. It can be delivered in terms of praising the user, and by other types of encouraging behavior. In contrast, task-related support consists of actions such as setting and reviewing log-in goals of eHealth interventions, positively reinforcing log-in and intervention use and providing answers to users on questions regarding the functionality of the eHealth solution.

We suggested that fairly simple non-responsive Embodied Conversational Agents (ECA’s) can provide a means for task-related support in order to make self-guided interventions a better experience. ECA’s are computer animations of faces or bodies, “robots on screen.” They can enrich the mostly text- and video-based self-guided eHealth interventions with an interface that has stronger similarities with a human face. Furthermore, they personify the interface and can contribute to a feeling of trust in the system (André and Pelachaud, 2010). ECA’s are applied within various contexts; from computer games (Bostan et al., 2009), intelligent tutoring systems (D’Mello et al., 2007), museum guides (Kopp et al., 2005) conducting medical interviews (Kobori et al., 2018), and providing therapy for depression and anxiety (Fitzpatrick et al., 2017).

Within all this ECA variety, we focus in this paper on ECA’s that take on the role of a learning coach or tutor within e-learning environments as (a) e-learning (psycho-education) is one of the cornerstones of self-guided eHealth interventions and (b) considerable progress in the application of ECA’s within the scientific domain of e-learning has been made, which has created a solid basis for further research.

### Current Evidence for ECA’s as Tutors Within e-Learning

Indeed, promising ECA effects have been found on e-learning. Within the meta-study of Schroeder et al. (2013) on 43 studies including 3,088 participants, a small but significant effect was reported on learning. The participants learned more from a system with a pedagogical agent, than a system without one. Next to learning, positive effects of ECA’s have been found on user motivation. The meta-study of Veletsianos and Russell (2014) reports on studies in which learner motivation and learning outcomes are promoted by pedagogical agents. However, the evidence is equivocal as their meta-study also refers to studies in which the pedagogical agents did not demonstrate added value compared to text-only conditions. They summarized these mixed results as a conundrum which is open for future research to resolve.

A research topic that often goes together with the effectiveness of pedagogical agents is that of the modalities (e.g., speech, animation) of the agents used. The relevance of the modalities for learning is expressed by the social cue hypothesis (Domagk, 2010) that states that the presence of social cues causes learners to engage in sense-making processes and processing the learning material deeply. Social cues as represented by the ECA’s modalities of e.g., visibility, speech and animation should -according to this hypothesis- have a positive impact on the learning process compared to a sheer textual environment.

### Effects of ECA Modalities of Speech and Visibility on e-Learning

Equal to the effects of ECA’s as a whole, the evidence for the effects of the ECA’s speech and visibility is mixed. Atkinson (2002) found that an ECA using speech performed better than an e-learning environment that lacked an ECA. This positive effect was replicated by Lusk and Atkinson (2007) and also Graesser et al. (2004) came to the same conclusion.

In contrast, Louwerse et al. (2005) report on studies in which pedagogical agents using speech had no additional effect compared to speech alone. Stated differently: those studies suggest that it is solely the speech that determines the learning effect and not the visual presence of the ECA. Schroeder (2013) found that speech-enabled ECA’s provide a better solution on learning measures than “ECA-less” learning environments. Schroeder therefore suggested that -contrary to the Louwerse et al. (2005) statement- the ECA’s visibility combined with their voice is more beneficial than voice alone.

A potential way to reconcile these conflicting results on the ECA’s visibility may be provided by the concept of distraction, which has also been described as the split-attention principle (Louwerse et al., 2005). According to this principle, users are hindered to engage themselves in the learning process, when they are obliged to simultaneously interact with an ECA. Van Mulken et al. (1998) found that ECA’s are indeed distracting, but also motivating.

Moreno and Mayer (2007) claim that the ECA’s motivation should be greater than their distraction to facilitate learning. We are unaware of studies on design guidelines for optimizing user motivation while minimizing user distraction. However, there are solutions to the attention split. An ECA can provide motivating instructions in advance of an e-learning topic, somewhat comparable to a traditional teacher in a classroom. Then, the user can be asked to start working on the topic, without further interference of the ECA. Consequently, the user can both dedicate their full attention to first the ECA and then to their own learning process.

Finally, a design argument in favor of a visible ECA, as a source for either speech or text, is provided by Cassell (2001). Cassell states that properly designed interfaces have affordances and visual clues that are in accordance with their role. Speech does not appear spontaneously; it therefore makes sense to present the ECA as its visible source.

### Effect of the ECA Modality of Animation on e-Learning

Technological advances have also made it easier to animate agents, instead of presenting them as still images. However, limited knowledge is available on whether these animations have advantages.

Baylor et al. (2003) investigated the effects of pedagogical agent speech (human, machine-generated) and animation (present, absent) on learning and motivation, Animation gave somewhat contradictory results: participants learned significantly more, but also reported that the agent was significantly less facilitative than when it was still. In addition, animation made the participants significantly less motivated about the topic. In contrast to these results, Schroeder et al. (2013) reported that still ECA’s produced a small but significant learning effect, whereas animated ECA’s neither produced a positive nor a negative effect.

### Expectations on ECA Research Within the Domain of Self-Guided eHealth Interventions

**FIGURE 1:**
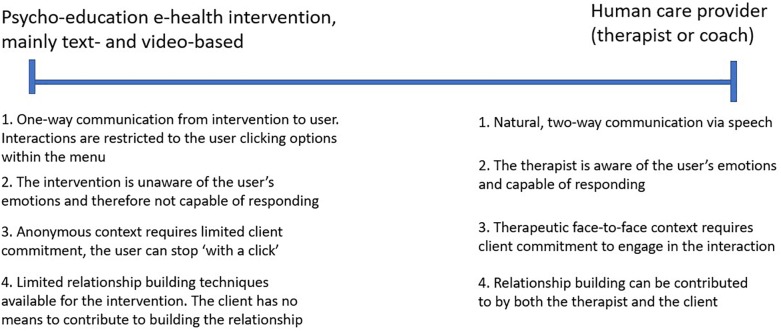
The left and right boundaries for ECA’s as adjuncts in e-mental Health.

In summary, the literature tells us that a visible speech-enabled or text-enabled ECA has e-learning benefits compared to no ECA at all. An important pre-condition is that the ECA will make a clear distinction between the moment it communicates to (or interacts with) the user and the moment they let the user learn. Whether an animated or still ECA is the better solution is open for further investigation. Within the context of this paper, we will concentrate on the e-learning domain within an e-mental health context, with patients as the targeted user group. Within this perimeter we will define what we can and should expect from an ECA. For this, please see the schematic picture of the research domain that we present below in Figure 1.

On the left side we place a classic self-guided eHealth intervention, such as MoodGym^1^ Within this type of intervention, the user is typically asked to read information and do exercises to improve their mental being. The intervention does not “see” or “hear” how the user is doing, will not understand the user and will therefore not be capable of expressing personal interest. On the right side we position an (idealized) human care provider who can and will interact with the user. He/she can hear and see the user, will take their emotions into account and respond appropriately by e.g., expressing empathy.

Within many experimental studies, ECA’s are set up with the intention to simulate processes that hinge to the right side of the spectrum. These ECA’s have the purpose of triggering social mechanisms that play a role within two-way human-to-human communication. Within this paper we opt for a different approach. Our aim is to find out whether we can make improvements on the left side: can we realize user experience improvements on a text- and video-based self-guided e-mental health intervention by adding an ECA that makes users more engaged and motivated? We chose this approach for the following reasons:

• Most evidence-based self-guided eHealth interventions are text- and video-based and unaware of users’ emotions. So, they are typical “left-side” interventions. If we want to improve adherence to the present base of eHealth solutions, and build upon the existing work done, we must start left.
• By separating the therapeutic content from the user support aspects, existing evidence-based self-guided eHealth interventions can remain unaltered. ECA’s can be added as adjuncts for providing directions and user support, without interfering with the functionality and evidence for the intervention.
• The ECA’s we envision are widely available. If we would be able to find a positive ECA effect, they can be easily implemented within web-based environments.

The aim of this study is to investigate the opportunities from within the technology to increase its support level toward the user. We will deploy a pedagogical agent acting as an adjunct to a self-guided positive psychology psycho-education intervention and investigate whether users will appreciate its task- and emotion-related support capabilities when compared to a text-only control condition.

## Materials and Methods

### Recruitment of Participants

We started the recruitment process by adding the experiment as an option to the university of Twente eHealth MOOC that is offered on the FutureLearn online course platform^2^. As the recruitment process of participants did not have the required pace, we decided to expand it. We recruited bachelor and master psychology students at the University of Twente. In total 230 participants were included. As an inclusion criterion we set a high level of mastery of English. As an exclusion criterion we set participation in a pre-study with the ECA. The study protocol was reviewed and approved by the Twente Institutional Review Board.

### Design

To investigate the differential outcome effects of ECA’s with different modalities as mentioned within the ECA literature, speech, visual presence of the ECA, and the level of animation, we set up conditions with the following distinctive ECA features:

• The ECA is animated (1) vs. the ECA is a still image (2) vs. the ECA is not visible (3).
• The ECA expresses itself via speech (1) vs. text (2).

Out of the six combinations, we left out animated, text (non-speech) as a key element of the ECA’s animation consisted of the lip sync which we would lose without speech. In addition, we left out the option not visible, speech as a voice without a visible source would create an unusual set-up.

This way, we created the following four conditions:

(1) AS = animated, speech (non-text),
(2) SS = still, speech (non-text)
(3) ST = still, text (non-speech)
(4) TO (control condition) = text-only

**FIGURE 2:**
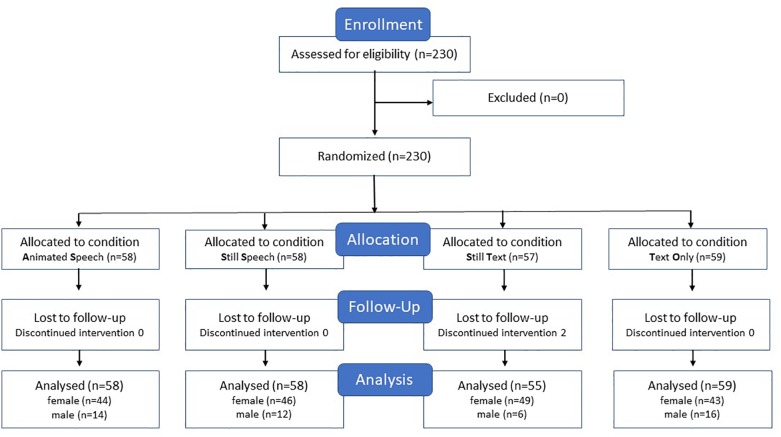
CONSORT flow chart for study participation.

The study design was a between-subjects experiment with the before-mentioned four conditions to which participants were randomly assigned using randomization software; AS (58 participants; 44 female, 14 male) SS (58 participants; 46 female, 12 male) ST (55 participants; 49 female, 6 male) TO (59 participants; 43 female, 16 male). Participants were on average 22.1 years of age and represented 16 nationalities of which German (70.1%) and Dutch (20.4%) were most prominent. See Figure 2 with further details.

### Intervention

An e-learning intervention for making people knowledgeable about positive psychology was set up. Positive psychology focuses on the abilities of people and their potential to flourish. Positive psychology was chosen for being a relevant topic within the eHealth domain; several treatments against depression are based on positive psychology principles (Hayes et al., 1999). In addition, positive psychology and happiness are subjects that are of general human interest. As we assumed, this would make it easier for participants to engage with our experimental set-up.

The self-guided intervention was developed by analyzing the positive psychology topic (Gable and Haidt, 2005) and creating a combination of theory and exercises, including the remunerated “three good things exercise^3^” and “best possible self-exercise” (Renner et al., 2014).

A WordPress website (version 4.9.7) ^4^ with four webpages was created, each representing a condition. The e-learning intervention on positive psychology was embedded as an online Microsoft PowerPoint presentation^®^ and placed on the left side on each of the four webpages. On the right side of the four webpages the user support content was added, as to represent the four conditions. The user support consisted of task-related support (e.g., “within this experiment you will read about positive psychology and you will do some exercises”) and emotional support (e.g., “well done!”). In addition, the user was stimulated to take advantage of the exercises outside of the experiment.

An explicit distinction was made between the instruction as delivered by either the ECA or the text-only control condition on the one hand and the user learning activities on the other hand. This was done to avoid the split-attention effect (Louwerse et al., 2005). During instruction on the right side of the webpage, the user was told what learning modules would come next. Then the user was asked to click on the left side of the webpage and do the e-learning. When the e-learning module had come to an end, the user was asked to go to the right side of the webpage for new instructions.

For the animated (AS) condition an ECA was created through the Voki application^5^, see Figure 2 below. For the other three conditions, a second Microsoft PowerPoint presentation^®^ was embedded on the right side of the page to which a still of the ECA had been added (SS and ST conditions) as well as speech fragments (ST condition) or textual information (ST and TO conditions).

### Procedure

The webpages were put online, and the study was run without human supervision to simulate the self-guided eHealth intervention context. Users were provided with an URL that led to the Qualtrics system^6^. A randomization software module redirected the users to one of four webpages. On the right side of the webpage, the users received instructions through the ECA or instructional PowerPoint. They were asked to do the reading of the Positive Psychology PowerPoint on the left side and then to come back to the instructional side of the page for following instructions. This way, the users received instructions, performed an experimental task, received positive feedback and new instructions. After the introduction, this cycle was repeated twice. Then the users were redirected from the WordPress website to the Qualtrics environment to fill in the questionnaires.

### Outcome Measures

For the outcome measures, a variety of scales was selected.

First, the EGameFlow scale (Fu et al., 2009) was selected, which measures learners’ enjoyment of e-learning games. The developers of this scale refer to the application of flow theory within education (Whalen and Csikszentmihalyi, 1991) and argue that the flow experience is a pre-condition for successful e-learning. Autonomy and feedback have been implemented in the EGameFlow scale to measure task-related support as provided by the Graphical User Interface (GUI) of the e-learning environment. This suited very well with the purpose of this experiment, in which there was an aim to measure the differential effects of ECA’s as task-related and emotion-related support providers. From both the feedback and autonomy scale three items were selected on the basis of validation and on the basis of the distinctive formulation of the questions. Both scales use a seven-point Likert scale ranging from “strongly disagree” to “strongly agree.” Within the wording of the subscales the word “game” was replaced by “online training.”

Next, the Instructional Materials Motivation Survey (IMMS) was selected. This scale measures students’ motivational reactions to self-directed instructional materials and is derived from the ARCS model (Keller, 1987, 2006), that has been applied to ECA’s in e-learning settings, e.g., (Shen, 2009). ARCS’ A refers to gaining and keeping the learner’s attention and stimulate their desire to learn. ARCS’ R is about making the instruction relevant to the learner’s personal experience, needs and goals. The attention (12 items) and relevance (9 items) scales both use a five-point Likert scale ranging from “not true” to “very true.”

Subsequently, Involvement was selected. The Personal Involvement Inventory (Zaichkowsky, 1994) is a context-free measure applicable to involvement with products, with advertisements, and with purchase situations. It has been applied before for measuring the effectiveness of environments with ECA’s (Lo and Cheng, 2010). It was selected for this experiment to measure user motivation in general. The scale consists of 10 items and uses a seven-point Likert scale with varying category names such as “appealing” vs. “not appealing” and “means nothing” vs. “means a lot to me.”

Last, the Rapport scale was selected. Rapport is an umbrella term for generic positive interactions between human counterparts, which as a term is also associated to terms as harmony, fluidity, synchrony and flow. Many studies have demonstrated that, when established, rapport facilitates a wide range of social interactions between humans including psychotherapy (Tsui and Schultz, 1985) teaching (Fuchs, 1987) and caregiving (Burns, 1984). Rapport has been used as an outcome measure in studies with users interacting with an ECA (Gratch et al., 2007). Advanced ECA’s that respond to the verbal and non-verbal behavior of the user in a contingent manner have indeed successfully created rapport. For this experiment’s non-responsive ECA, we didn’t expect effects of rapport, but the outcome measure was added for exploratory and verification purposes. The Rapport scale (Cerekovic et al., 2014) consists of fifteen items and we used a seven-point Likert scale ranging from [(1) – Disagree strongly to (7) – Agree strongly].

## Analysis

### Visibility, Speech and Animation as ECA Modality Features

As a first step, conditions on common features were categorized. The AS, SS and ST conditions were put together in the Visible ECA category (171 participants) and compared to the Non-visible category (59 participants) that solely consisted of the TO condition. Furthermore, the AS and SS conditions were put together in the Speech ECA category (116 participants) and were compared to the Text category (114 participants) that consisted of both the ST and TO condition. The rapport outcome variable was only measured for the ST condition. Last, the AS represented the Animated ECA category (58 participants) and was compared to the Non-Animated ECA category (113 participants) that consisted of the SS and ST conditions. Obviously, the TO condition was not part of this analysis as it did not contain a visible ECA. We used a two-way analysis of variance (two-way ANOVA) to calculate differential effects between the modality features and to calculate interaction effects on the ECA’s feature and the gender of the participant. Although, prior to the analysis, we did not expect that gender would have an effect, a pre-analysis on gender showed differently.

### Four Conditions

Last, a two-way analysis of variance (ANOVA) on the four non-categorized conditions and their interaction with gender was conducted. This was done to look for effects of combinations of modalities, where combinations could be stronger (or less strong) than the individual modality effects. Additionally, Tukey *post hoc* tests were performed to look out for significant differences between individual conditions in combination with gender type.

## Results

### Visible vs. Non-visible ECA

The means, 95% Confidence Interval and SD values of all outcome variables are shown in Table 1.

**Table 1:** Mean scores and standard deviation on the visibility- non-visibility distinction.

	Visible ECA			Non-visible ECA		
	Female	Male	All	Female	Male	All
	(*n* = 139)	(*n* = 32)	(*n* = 171)	(*n* = 43)	(*n* = 26)	(*n* = 59)
Feedback (1-7)	4.5 (4.3-4.7; 1.2)**	4.9 (4.5-5.3; 1.0)**	4.7 (4.5-4.9; 1.2)*	4.5 (4.1-4.9; 1.2)**	4.0 (3.4-4.6; 1.0)**	4.2 (3.9-4.6; 1.1)*
Autonomy (1-7)	5.4 (5.2-5.6; 0.9)	5.7 (5.3-6.0; 0.8)	5.5 (5.3-5.7; 1.0)**	5.3 (5.0-5.6; 1.0)	5.0 (4.5-5.5; 1.0)	5.1 (4.8-5.4; 1.0 )**
Attention (1-5)	3.7 (3.6-3.8; 0.6)	3.6 (3.4-3.9; 0.5)	3.7 (3.6-3.8; 0.6)	3.7 (3.5-3.9; 0.6)	3.5 (3.2-3.8; 0.6)	3.6 (3.4-3.8; 0.6)
Relevance (1-5)	3.6 (3.5-3.7; 0.7)	3.5 (3.3-3.8; 0.6)	3.6 (3.5-3.7; 0.7)	3.8 (3.7-4.0; 0.6)	3.5 (3.2-3.9; 0.5)	3.7 (3.5-3.9; 0.6)
Involvement (1-7)	5.3 (5.1-.5; 1.1)	5.3 (4.9-5.7; 1.1)	5.3 (5.1-5.5; 1.1)	5.3 (5.0-5.7; 1.0)	5.1 (4.6-5.6; 0.6)	5.3 (4.9-5.5; 0.9)
Rapport (1-5)	4.8 (4.7-5.0;0.7 )	4.7 (4.5-5.0;0.7 )	4.8 (4.7-4.9; 0.7 )	n.a.	n.a.	n.a.


*^∗^significant effect of p = 0.03, ^∗∗^significant effect of p = 0.02.*

Comparing the visible and non-visible ECA, significant main effects were found on the outcome variables feedback^∗^ (*F* = 4.64; *p* = 0.03), and autonomy^∗∗^ (*F* = 5.17; *p* = 0.02); in both cases the visible ECA category resulted in significantly higher scores than the non-visible ECA.

**FIGURE 3:**
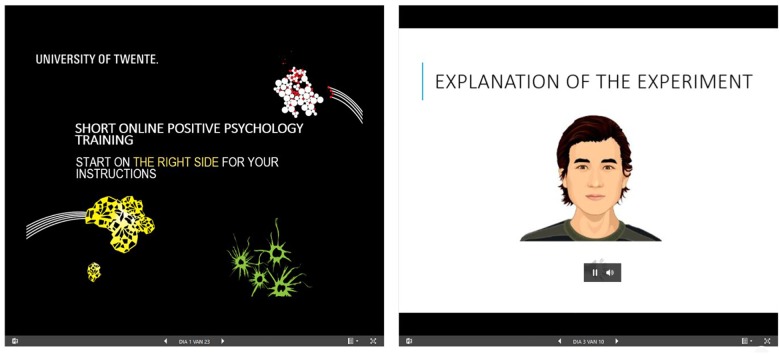
The e-learning intervention. On the left side of the webpage the educational content is displayed, on the right side the support condition with directions (task-related support) and encouragement (emotion-related support) is presented. The example shown is the AS condition; animated, speech.

No significant main effects were found for the other outcome variables: attention (*F* = 0.65, *p* = 0.42), relevance (*F* = 1.14, *p* = 0.29), involvement (*F* = 0.15, *p* = 0.70). Subsequently, the interaction between the visibility distinction and gender type was analyzed. A significant interaction effect between visibility^∗^gender was found for the outcome variable feedback^∗∗^ (*F* = 5.26, *p* = 0.02). The interaction effect is visually presented in Figure 3 below; male participants graded the *visible* ECA *higher* than female participants but graded the *non-visible* ECA *lower* than female participants.

Contrary to the feedback outcome variable, for the autonomy outcome variable a significant interaction effect between visibility and gender was *not* found (*F* = 2.92, *p* = 0.09) within the two-way ANOVA. In addition, the two-way ANOVA showed that no significant interaction effects with gender were found for the other outcome variables; attention^∗^gender: (*F* = 0.86, *p* = 0.36), relevance^∗^gender (*F* = 1.21, *p* = 0.27), involvement^∗^gender (*F* = 0.45, *p* = 0.50).

### Text vs. Speech

The means, 95% Confidence Interval and SD values of the distinction of an ECA that communicates via speech or text, are shown in Table 2 below.

**Table 2:** Mean scores and standard deviation on the speech-text distinction.

	Speech			Text		
	Female	Male	All	Female	Male	All
	(*n* = 90)	(*n* = 26)	(*n* = 116)	(*n* = 92)	(*n* = 22)	(*n* = 114)
Feedback (1–7)	4.5 (4.3–4.8; 1.2)	5.0 (4.5–5.4; 1.3)	4,7 (4.5–5.0; 1.2)*	4.4 (4.2–4.7; 1.2)	4.2 (3.7–4.7; 1.0)	4,3 (4.0–4.6; 1.1)*
Autonomy (1–7)	5.4 (5.2–5.6; 1.0)	5.7 (5.3v6.0; 0.9)	5.5 (5.3–5.7; 1.0)	5.4 (5.2–5.6; 1.0)	5.7 (4.7–5.6; 0.9)	5.3 (5.0–5.5; 1.0)
Attention (1–5)	3.7 (3.6–3.8; 0.6)	3.7 (3.4–3.9; 0.4)	3.7 (3.6–3.8; 0.6)	3.7 (3.6–3.8; 0.6)	3.5 (3.2–3.7; 0.5)	3.6 (3.4–3.7; 0.6)
Relevance (1–5)	3.6 (3.5–3.8; 0.6)	3.6 (3.3–3.8; 0.4)	3.6 (3.5–3.7; 0.6)	3.7 (3.6–3.8; 0.7)	3.5 (3.3–3.8; 0.7)	3.7 (3.5–3.8; 0.7)
Involvement (1–7)	5.2 (5.0–5.4; 1.1)	5.4 (4.9–5.8; 1.0)	5.3 (5.1–5.5; 1.1)	5.4 (5.2–5.7; 1.1)	5.0 (4.1–5.9; 0.8)	5.3 (5.0–5.5; 1.0)
Rapport (1–5)	4.9 (4.8–5.0; 0.1)	4.7 (4.4–4.9; 0.1)	4,8 (4.6–4.9; 0.1)	4.8 (4.6–5.0; 0.1)	5.0 (4.5–5.6; 0.3)	4,9 (4.6–5.2; 0.2)


*^∗^significant effect of p = 0.02.*

A significant effect on feedback^∗^ (*F* = 5.32, *p* = 0.02) was found; speech led to significantly higher scores than text. For the other variables no significant effects were found; autonomy (*F* = 2.40, *p* = 0.12), attention (*F* = 1.19, *p* = 0.28), relevance (*F* = 0.00, *p* = 0.96), involvement (*F* = 0.10, *p* = 0.75), rapport (*F* = 0.39, *p* = 0.54). Subsequently, the interaction between the speech-text category and gender type was analyzed. No significant interaction effects were found. Feedback^∗^gender (*F* = 3.29, *p* = 0.07), autonomy^∗^gender (*F* = 2.36, *p* = 0.13), attention^∗^gender: (*F* = 0.79, *p* = 0.38), relevance^∗^gender (*F* = 0.18, *p* = 0.68), involvement^∗^gender: (*F* = 2.07, *p* = 0.15), rapport^∗^gender (*F* = 2.14, *p* = 0.15).

### Animation vs. no Animation as Categories

Subsequently, the effect of the modality of animation was analyzed. The means, 95% Confidence Interval (CI) and SD values of the distinction of an ECA that is animated or still, are shown in Table 3 below.

**Table 3:** Mean scores, CI and standard deviation on the animated-still distinction.

	Animated ECA			Still ECA		
	Female	Male	All	Female	Male	All
	(*n* = 44)	(*n* = 14)	(*n* = 58)	(*n* = 95)	(*n* = 18)	(*n* = 113)
Feedback (1-7)	4.3 (3.9-4.7;1.2)	5.1 (4.5-5.8; 1.2)	4.5 (4.2-4.8; 1.3)	4.5 (4.3-4.8; 1.2)	4.7 (4.2-5.3; 1.2)	4.6 (4,3-4.8; 1.2)
Autonomy (1-7)	5.3 (5.0-5.6; 1.0)	5.8 (5.3-6.3;0.9)	5.4 (5.2-5.7; 0.97)	5.4 (5.2-5.6; 1.0)	5.5 (5.1-6.0; 0.8)	5.5 (5.3-5.6; 1.0)
Attention (1-5)	3.7 (3.5-3.8; 0.7)	3.6 (3.3-3.9; 0.5)	3.6 (3.5-3.8; 0.6)	3.7 (3.6-3.8; 0.6)	3.7 (3.4-4.0; 0.4)	3.0 (3.6-3.8; 0.6)
Relevance (1-5)	3.6 (3.4-3.8; 0.6)	3.6 (3.2-3.9; 0.3)	3.6 (3.4-3.7; 0.6)	3.6 (3.5-3.7; 0.7)	3.54 (3.3-3.9; 0.7)	3.6 (3.5-3.7; 0.7)
Involvement (1-7)	5.1 (4.8-5.4; 1.2)	5.2 (4.6-5.8; 1.1)	5.1 (4.8-5.4; 1.1)	5.4 (5.2-5.6; 1.1)	5.4 (4.8-5.9; 1.1)	5.4 (5.2-5.6; 1.1)
Rapport (1-5)	4.8 (45.0-5.6; 0.7)	4.7 (4.4-5.1; 0.8)	4.8 (4.6-5.0; 0.7)	4.9 (4.7-5.0; 0.7)	4.73 (4.4-5.1; 0.7)	4.8 (4.7-5.0; 0.7)


No significant effects of animation on any of the outcome variables was found: feedback (*F* = 0.14; *p* = 0.71), autonomy (*F* = 0.13; *p* = 0.72), attention (*F* = 0.24, *p* = 0.62), relevance (*F* = 0.00, *p* = 0.95), involvement (*F* = 0.92, *p* = 0.34), rapport (*F* = 0.01, *p* = 0.91).

No significant interaction effects between level of animation and gender type were found either. feedback^∗^gender (*F* = 1.42, *p* = 0.24), autonomy^∗^gender (*F* = 1.30, *p* = 0.26), attention^∗^gender (*F* = 0.03, *p* = 0.86), relevance^∗^gender (*F* = 0.05, *p* = 0.83), involvement^∗^gender (*F* = 0.17, *p* = 0.68), rapport^∗^gender (*F* = 0.12, *p* = 0.91). However, a gender effect on the variable feedback (*F* = 4.15, *p* = 0.04) was found. Male participants graded the ECA significantly higher that female participants. The reason this gender effect for feedback was solely found in the animation-still analysis is due to the text-only scores that were out of scope. This contrasts with the speech-text and visibility-non-visibility analyses for which text-only scores were in scope. Gender effects were not found for the other outcome variables; autonomy (*F* = 2.57, *p* = 0.11), attention (*F* = 0.11, *p* = 0.74), relevance (*F* = 0.11, *p* = 0.74), involvement (*F* = 0.05, *p* = 0.82), rapport (*F* = 0.65, *p* = 0.42).

### Effects of Individual Conditions

Last, the individual conditions were analyzed to look for differences between combinations of modalities. The means, 95% Confidence Interval and SD values of the distinction of an ECA is animated, communicates via speech or text, and is visually present or not are shown in Table 4 below.

**Table 4:** Mean scores and standard deviation of the four conditions.

	AS	SS	ST	TO
Feedback (1-7)	4.5 (4.2–4.8; 1.3)	4.7 (4.4–5.0; 1.2)	4.4 (4.1–4.7; 1.1)	4.4 (4.1–4.7; 1.1)
Autonomy (1-7)	5.4 (5.2–5.7; 1.0)	5.4 (5.2–5.7; 0.9)	5.5 (5.2–5.7; 1.0)	5.2 (5.0–5.5; 1.0)
Attention (1-5)	3.6 (3.5–3.8; 0.6)	3.7 (3.6–3.9; 0.6)	3.6 (3.5–3.8; 0.6)	3.6 (3.5–3.8; 0.6)
Relevance (1–5)	3.6 (3.4–3.7; 0.6)	3.7 (3.5–3.8; 0.6)	3.5 (3.4–3.7; 0.8)	3.8 (3.6–3.9; 0.6)
Involvement (1-7)	5.1 (4.9–5.4; 1.1)	5.4 (5.1–5.6; 1.0)	5.4 (5.1–5.7; 1.2)	5.3 (5.0–5.6; 0.9)
Rapport (1-5)	4.8 (4.6–5.0; 0.7)	4.9 (4.7–5.1; 0.7)	4.8 (4.6–5.0; 0.7)	n.a.


No significant effects of the conditions on the outcome variables were found; feedback (*F* = 1.73; *p* = 0.16); autonomy (*F* = 1.70; *p* = 0.17), attention (*F* = 0.59, *p* = 0.62), relevance (*F* = 0.52, *p* = 0.67), involvement (*F* = 0.49, *p* = 0.69), rapport (*F* = 0.21, *p* = 0.81). However, *post hoc* Tukey tests on the individual conditions revealed significant differences for the autonomy outcome variable between AS, the most feature rich condition and TO (*p* = 0.04), the control condition. For the feedback outcome variable, the differences between AS and TO (*p* = 0.05) and SS and TO (*p* = 0.05) both reached significance. Subsequently, the interaction between the four conditions and gender type was analyzed. For mean scores, 95% Confidence Interval and SD values, see Table 5 below.

**Table 5:** Mean scores and standard deviation of the four conditions^∗^gender type.

	AS		SS		ST		TO	
	Female	Male	Female	Male	Female	Male	Female	Male
	(*n* = 44)	(*n* = 14)	(*n* = 46)	(*n* = 12)	(*n* = 49)	(*n* = 6)	(*n* = 43)	(*n* = 16)
Feedback (1-7)	4.3 (4.0-4.7; 1.2)	5.1 (4.5-5.7; 1.2)	4.7 (4.4-5.1; 1.2)	4.8 (4.1-5.5; 1.4)	4.4 (4.0-4.7; 1.2)	4.7 (3.7-5.6; 0.9)	4.5 (4.1-4.9; 1.2)	4.0 (3.4-4.6; 1.0)
Autonomy (1-7)	5.3 (5.0-5.6; 1.0)	5.8 (5.3-6.3; 0.9)	5.5 (5.2-5.7; 1.0)	5.5 (4.9-6.0; 0.9)	5.4 (5.2-5.7; 1.1)	5.7 (4.9-6.5; 0.7)	5.3 (5.0-5.6; 1.0)	5.0 (4.5-5.5; 1.0)
Attention (1-5)	3.7 (3.5-3.8; 0.7)	3.6 (3.3-3.9; 0.5)	3.7 (3.6-3.9; 0.6)	3.8 (3.4-4.1; 0.4)	3.7 (3.5-3.8; 0.6)	3.5 (3.0-4.0; 0.6)	3.7 (3.5-3.9; 0.6)	3.4 (3.2-3.8; 0.6)
Relevance (1-5)	3.6 (3.4-3.8; 0.6)	3.6 (3.2-3.9; 0.3)	3.7 (3.5-3.9; 0.7)	3.6 (3.2-3.9; 0.5)	3.5 (3.4-3.7; 0.7)	3.5 (3.0-4.0; 1.1)	3.9 (3.6-4.0; 0.6)	3.6 (3.2-3.9; 0.5)
Involvement (1-7)	5.1 (4.8-5.4; 1.2)	5.2 (4.7-5.8; 1.1)	5.3 (5.0-5.6; 1.0)	5.6 (4.9-6.1; 1.0)	5.5 (5.2-5.7; 1.1)	5.0 (4.1-5.8; 1.3)	5.3 (5.0-5.7; 1.0)	5.1 (4.6-5.6; 0.6)
Rapport (1-5)	4.8 (4.6-5.0; 0.7)	4.7 (4.4-5.1; 0.8)	5.0 (4.8-5.2; 0.7)	4.6 (4.2-5.0; 0.7)	4.8 (4.6-5.0; 0.7)	5.0 (4.5-5.6; 0.7)	n.a.	n.a.


No significant effects of the interaction between the conditions and gender type were found on any of the outcome variables feedback^∗^gender (*F* = 2.29, *p* = 0.79), autonomy^∗^gender (*F* = 1.47, *p* = 0.22), attention^∗^gender: (*F* = 0.35, *p* = 0.79), relevance^∗^gender (*F* = 0.40, *p* = 0.75), involvement^∗^gender: (*F* = 0.71, *p* = 0.54), rapport^∗^gender (*F* = 1.46, *p* = 0.23).

However, *post hoc* Tukey tests with selections on male participants on AS vs. TO as control condition showed significant effects on feedback (*t* = 2.81, *p* = 0.01) and autonomy (*t* = 2.54, *p* = 0.02). The *post hoc* Tukey tests on gender differences for AS showed that for feedback male participants (5.12) graded it significantly higher (*t* = 2.06, *p* = 0.04) than female participants (4.34). No other significant effects were found in the Tukey *post hoc* test.

## Discussion

### Principal Results

Within this study we found that visibility of the ECA does have a positive effect on the outcome measures of feedback and autonomy. Furthermore, on feedback we found a gender effect. Male participants graded the *visible* ECA *higher* than female participants and graded the *non-visible* ECA *lower* than female participants. This feedback effect was corroborated by gender analyses on animation and on the separate conditions, where male participants scored the ECA significantly higher than female participants. Speech communication by the ECA also had a positive effect on feedback, without differentiating between gender type. Animation did not show effects in this study.

### Interpretation of the Nature of the Outcome Variables

**FIGURE 4:**
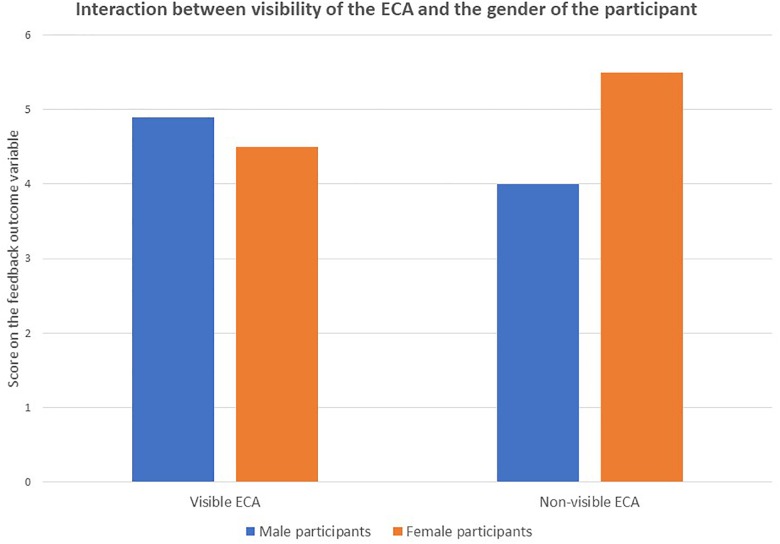
Interaction effect of visibility^∗^gender type for the feedback variable.

When interpreting these results, one of our first questions was: why were effects found on feedback and autonomy and not on the other outcome variables? We suspected that the nature of the outcome variables could play a role. As they measured different constructs, we decided to analyze their specific character and purpose in relation to our results. Figure 4 below depicts our experimental outcome variables, which we ranked according to the level of abstraction.

**FIGURE 5:**
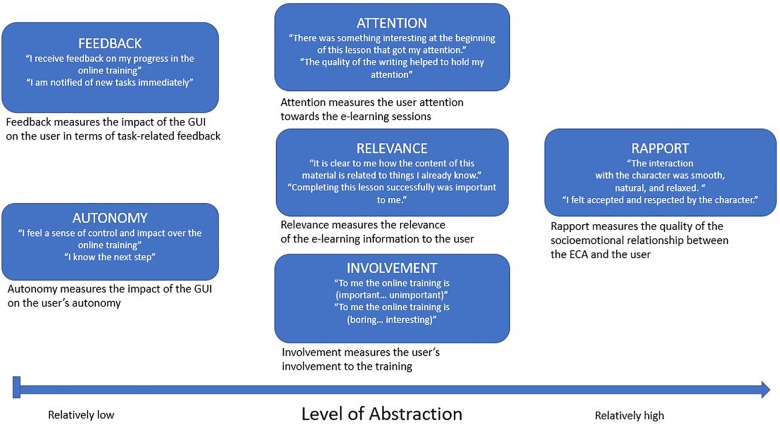
Sequence order of the outcome variables in terms of level of abstraction.

As Figure 5 shows, the task-related outcome variables feedback and autonomy are ranked lowest on level of abstraction. We will further discuss the figure, going from left to right.

### Feedback and Autonomy During the Online Training

These constructs address the way the GUI presents the user’s tasks. The feedback and autonomy results demonstrate that when a user is doing the experiment, task-related support is more effective when delivered by a visible and speech-enabled ECA than by mere text. The social cue hypothesis that predicts deeper processing and higher personal relevance is therefore applicable to the modalities of visibility and speech, but not to animation. This is in accordance with the experimental result on animation of Lusk and Atkinson (2007) and with the stance that animation engages but also distracts users (Moreno et al., 2000).

The engagement effect of animation seems to fit better with emotion-related support than with task-related support. However, our experiment did not demonstrate emotion-related effects of any kind, which we will discuss below in relation to the user state of distress. The explanation for the lack of an animation effect is further complicated by gender type; male participants graded animation significantly higher than female participants. This may be explained as a gender resemblance effect, (Baylor, 2011) but deserves further research.

### Attention and Relevance and Involvement With the Training

Attention, Relevance and Involvement question the user’s learning experience. On these outcome variables, the visible and speech-enabled ECA did not induce effects. We interpret this as: although the users appreciated the feature-rich ECA providing task-related support (as demonstrated by the effects for feedback and autonomy), this effect did not *transfer* to the learning experience. In addition, the social cue hypothesis is not applicable to these outcome variables. We will expand on the reasons why this may be, further below.

### Rapport With the ECA

Most abstract is rapport, the relationship outcome variable. Rapport was measured on the three ECA conditions and not on the text-only condition. It measures the extent to which a relationship has been built between user and ECA. We added the variable for observation purposes. That is, we reckoned that it would be ambitious to expect signs of a relationship after a 30 min experiment where long-term interactions of e.g., 30 days are advised (Bickmore and Picard, 2005). The outcome was somewhat different from what we expected: rapport reached a fairly elevated level (e.g., see Table 1, Rapport score reached 4.8 on a scale from 1–7), but there were no differences amongst the conditions. Establishing a relationship is one thing, maintaining a user-ECA relationship for a longer period is challenging as is exemplified by the quote of Bickmore et al. (2010) on a mundane ECA characteristic “It would be great if Laura could just change her clothes sometimes.”

### Comparison of Our Results to Prior ECA Studies and Theories

Summarizing the results on the outcome variables, we found partial effects on feedback and autonomy. These constructs measure task-related support as provided by the GUI. No effects on learning experience and motivation were found, contrary to the results of the review study of Schroeder et al. (2013). The implication of the social cue hypothesis of “the more social cues, the more social effects” was therefore only partially confirmed. However, in line with the results of Schroeder et al. (2013) we found an effect of the visibility of the ECA, but on a non-learning outcome variable: feedback. The feedback effect is in accordance with our expectation that users value practical support (*task-related support*) such as positively reinforcing log-in and intervention use when delivered by a simple, non-responsive ECA. This result for the feedback outcome variable also fits with Cassells’s (2001) affordances that a visible ECA adds value as to make explicit *who* delivers the support. The *emotion-related support* of the ECA (positive confirmation after a lesson was done) seemed to have no effect on the learning experience.

### Support Is Potentially Only Needed When in Distress

The question is why the experiment did not show an ECA effect on learning experience. The answer may be found within the qualitative remarks of the participants, that generically stated the experiment was a pleasant task to do. These remarks and the fact that the participants were mainly psychology students that are familiar with the positive psychology learning concepts, make it unlikely that a need for emotional support was induced. This probably made the social cues of the ECA superfluous. We further reason that users that experience episodes of distress (such as eHealth patients dealing with serious issues) have a greater need and indeed appreciation for support (Kraft et al., 2008).

We envision a follow-up experiment during which users will carry out a mentally fatiguing pre-task, after which the effects of a supportive ECA will be assessed again. This concept is in line with the strength model (SM), a theory that describes that all acts of self-regulation rely on a common and limited energy source (Baumeister et al., 2007). According to this view, self-regulatory effort drains energy and leads to ego depletion (Baumeister et al., 2018) for which emotional support can provide a remedy (Kraft et al., 2008).

### Additional Measurement Instruments

We started out by stating that ECA studies in general provide enigmatic results. Our results fit within this overall picture of ECA research. As an explanation, questionnaires as research tools may have their limitations measuring what users do and decide when interacting with ECA’s. We envision a pre-experimental phase, during which users will shortly interact with both a text-only interface and an ECA interface. As a next step the user will be asked to choose their preferred interface for the core experiment. We wonder whether users will demonstrate a slight preference for ECA’s compared to text-only solutions (as the present results suggest) or whether other results will appear. By continuing to use questionnaires at the end of the experiment, we may be able to cross-validate the users’ prior decisions.

Last, a remarkable result of our experiment is the gender effect that we found. Male participants valued our (male) ECA better in terms of feedback. We suspect this is an effect of gender identification, but it deserves further investigation. If we elaborate the action-driven method outlined above with a female ECA option, we will be able to test whether female participants choose female ECA’s and whether they will score them higher on animation than they did within this experiment.

### Limitations

Conclusions on ECA research are in general limited to their task and context. Concerning the task and context that were specific to our experimental set-up and could have influenced our results, we separated learning content (left part of the screen) from supportive content (right part of the screen). In addition, as learning content we used a positive psychology intervention. As supportive content we provided directions and gave positive feedback after a learning task was finalized by the user. This way we avoided distraction from the ECA toward the user, but we are not aware of similar set-ups in real life. The supportive content could be controlled by the user by using the click-through buttons, which provided user control, but which is unlike some other ECA set-ups that use vocal user input. Our intervention was a short-term, one-off intervention. It is not known how this can be translated to life interventions that typically span a period of 6–10 weeks and are used on a more frequent basis. Our feedback and autonomy outcome measures were both restricted to three items, more items would have been welcome. Our participants were likely in a mental state of limited or no stress, which most likely did not induce a need for support. Furthermore, our participants were psychology students with a high mastery of English of which it is unsure how well they represent the eHealth user population.

## Conclusion

Our experiment showed positive ECA effects when providing task-related support to users of a psycho-education environment. The ECA as a GUI seemed to make the task easier than text. However, our ECA was not capable of demonstrating effects because of its emotion-related support. This may be due to the friendly set-up of our experiment, that failed to bring users to a distressed, need-for-support mental state. Our hypothesis is that this disguises the true supportive potential of ECA’s. Future research should aim to experimentally bring users to a mentally fatigued state within a long-term intervention to investigate whether emotional ECA support can be effective for user motivation. If indeed the ECA proves to be useful for users in such conditions, this provides a valuable argument for adding non-responsive ECA’s to self-guided eHealth interventions for the sake of higher adherence and effect.

We reckon that Figure 1, describing a continuous line from support by the technology to support by human care providers, is relevant within the eHealth context. Our stance is that “right-side” human support has its unique merits with which ECA’s should not compete. The fact of the matter is that self-care technology has more potential than just providing tasks to users. The technology can be endowed with task-related and emotion-related supportive features from which users of self-guided interventions can benefit. We should not miss the opportunity to inform the “left-side” technology to the support needs of our patients. To realize this, we can add ECA’s as a visible source of either supportive textual or (preferably) speech messages. In case we become successful at realizing support from within the technology itself, users of self-guided interventions will likely demonstrate higher adherence.

## Ethics Statement

This study was carried out in accordance with the recommendations BMS Ethics Committee of the university of Twente with informed consent from all subjects. All subjects gave informed consent in accordance with the Declaration of Helsinki. The protocol was approved by the BMS Ethics Committee of the university of Twente.

## Author Contributions

All authors conceived and designed the study, analysis and/or interpreted the data, and critically revised the manuscript for important intellectual content. MS acquired the data and drafted the manuscript. SK and JVG-P approved the final version of the manuscript to be published.

## Conflict of Interest Statement

The authors declare that the research was conducted in the absence of any commercial or financial relationships that could be construed as a potential conflict of interest.

## Footnotes

1 https://moodgym.com.au/

2 https://www.futurelearn.com/

3 https://ggia.berkeley.edu/practice/three-good-things

4 https://wordpress.org

5 https://www.voki.com

6 https://www.qualtrics.com
